# Supplementary material for: Are carbon emissions trading and green financial instruments synergistic? -Comprehensive quantitative research based on content analysis
Source: PLoS One. 2024 Mar 7;19(3):e0298601. doi: 10.1371/journal.pone.0298601 (PMC10919601; doi:10.1371/journal.pone.0298601)
Supplement: S1 Data — (ZIP) [file pone.0298601.s001.zip › Data/地级市Z_F工作报告.zip!/║╙─╧/Σ≡║╙╩╨/└┤╫╘╣1⁄2╓┌║┼í╛┬φ┐╦╩2╛▌═°í┐_2011.docx]

各位代表:

现在，我代表市人民政府，向大会作政府工作报告，请予审议，并请各位政协委员提出意见。

折叠编辑本段一、2010年及"十一五"工作回顾

2010年，面对复杂多变的发展形势，市政府在省委、省政府和市委的正确领导下，在市人大、市政协的监督支持下，团结带领全市人民，以科学发展观统领全局，主动把握机遇，努力克服困难，奋力开拓创新，圆满完成了市五届人大五次会议确定的目标任务，实现了"十一五"规划的精彩结局。

一年来,我们重点抓了以下工作:

(一)牢牢把握发展第一要务，着力推进经济提速提效。我们咬定发展不放松，改革创新破难题，立足当前保增长，着眼长远促转型，致力提升发展质量和效益。全市生产总值682亿元、比上年增长14.7%，增速居全省第二位;财政总收入55亿元、净增11亿元、增长25.2%，地方财政一般预算收入26.1亿元、净增5.7亿元、增长28%，增幅居全省第二位，是近十年收入增量最多的一年;税收收入占地方财政一般预算收入的比重达到82.3%、居全省第一位;规模以上工业完成增加值395亿元、增长20.3%，完成销售收入1635亿元，实现利税264.6亿元，分别增长34.9%和41.4%;全社会固定资产投资404亿元、增长24.7%;社会消费品零售总额216.3亿元、增长18.4%;城镇居民人均可支配收入14769元、增长10.3%，农民人均纯收入6460元、增长14.9%，城乡居民储蓄存款余额260.3亿元，全市金融机构新增贷款56亿元，是近十年信贷投放最多的一年。

(二)积极承接产业转移，着力推进招商引资和重点项目建设。始终把招商引资作为推动漯河发展的"一号工程"，扎实开展招商引资"百日竞赛"，高水平举办第八届中国(漯河)食品博览会、第九届中部糖酒会、第三届豫菜品牌大赛，持续掀起招商引资热潮。全年实际利用外资3.2亿美元，实际利用省外资金85亿元,新签约各类项目519个，投资总额1060亿元，合同履约率88.8%，我市荣获全省对外开放优秀市称号。始终把投资作为第一拉动力，强力推进重点项目建设，市定100个重点项目完成投资138.5亿元，新上项目开工率达到94%，总投资25亿元的华电漯河热电、总投资10亿元的太古可口可乐等47个项目竣工投产。纳入全省转型升级"双千工程"的59个项目开工率居全省首位，投资完成率居全省第二位。

(三)加快发展现代农业，着力推进农业增效农民增收。坚持不懈做好"三农"工作，优化农业结构，扎实开展粮食产量、品质"双提升行动"，深入实施"双汇产业化工程"，农业产业化经营水平明显提高。全市优质粮食种植面积占总播种面积的比例达到91.4%，粮食总产达到168万吨，夏粮总产增幅全省第一。秸秆综合利用率达到86%。新增旱涝保收田面积3万亩。各类农业产业化龙头企业达到445家，农业产业化经营组织发展到2620家，新培育现代农业示范园区20个，标准化规模养殖场(小区)达到518个，生猪、蛋鸡、肉鸡规模饲养比重均居全省前列。

(四)紧紧围绕产业集聚集约发展，着力推进产业集聚区建设。坚持"三规合一"、产城融合、集约集聚要求，完善规划布局，强化基础投入，加强扶持引导，加快项目进驻，开展观摩点评，促进产业集聚区蓬勃发展。六大产业集聚区完成投资220亿元，累计入驻企业664家，完成主营业务收入800亿元，我市连续获得全省重点项目和产业集聚区观摩活动豫东南片区第一名。经济开发区升格为国家级经济技术开发区。

(五)全面加强城乡建设和管理，着力推进城乡统筹发展。认真做好城乡规划编制，不断加快城乡基础设施建设。全市城镇化率达到40.9%，提高1.7个百分点。高标准推进沙澧河开发建设，沙河一期竣工开园，沙河二期开工建设，沙澧河风景区成为国家3A级旅游景区。漯河新区获得批准，西城区建设全面启动。旧城区、城中村改造稳步推进，市区9条主干道升级改造和一批背街小巷改造成效明显，供热、供气、供水、垃圾处理、污水处理等城市基础设施建设步伐加快。狠抓市容市貌整治，重拳治理违法建设，城市管理水平明显提高。加快农村基础设施建设，新建成35个综合示范村和一批农村新型社区，新建改造农村公路246公里，新解决14.8万人的饮水安全问题，沼气普及率达到40%，新建村级邮站600个。全面实施"乡村清洁工程"，完成215个村的基础设施建设和村容村貌整治，农村生产生活条件明显改善。

(六)始终坚持以民为本，着力推进民生改善和社会事业繁荣。坚持把就业作为保障民生的首要任务来抓，全民创业和就业再就业工作扎实推进，城镇登记失业率低于省控目标1.96个百分点。进一步完善社会保障体系，城镇职工养老保险参保率、城镇居民医疗保险参保率、新型农村合作医疗参合率均达到98%;企业离退休人员退休金实现按时足额发放，新型农村社会养老保险试点顺利推进，10万名六十岁以上的农民按月领取基本养老保障金;城乡低保金及时足额发放，城乡困难群众基本生活得到保障;农村五保对象集中供养率达67%，居全省前列;开工建设经济适用房35万平方米、廉租房27万平方米，发放住房公积金贷款3.7亿元，帮助2589户职工家庭改善了住房条件。各级财政投入40.5亿元用于社会事业建设，省定、市定民生工程全面完成。认真落实义务教育"两免一补"政策，漯河医专新校区建设加快推进，职业教育"双十工程"扎实有效。城乡医疗卫生服务体系不断完善。建设27个乡镇综合文化站和234个农家书屋，许慎文化园建成开放，第二届许慎文化国际研讨会成功举办，中华经典文化诵读活动叫响全国。积极承办中国国际女排精英赛，在省十一届运动会上取得团体总分第五名的好成绩。科技工作得到加强，人才作用有效发挥。丹江口库区一期移民安置任务圆满完成，对口援建工作走在全省前列。计划生育、环境保护和土地管理三大国策得到较好落实，全省循环经济试点市建设扎实推进，节能减排目标全面完成。食品药品安全监管工作成效明显，获得全省食品安全优秀城市称号。信访工作全省先进，安全生产形势总体平稳，公众安全感指数全省第一，社会大局和谐稳定。

加强民主法制建设，自觉接受人大和政协监督，认真办理人大代表建议和政协委员提案。深入推进反腐倡廉建设。加强精神文明建设，公民道德水平和文明程度不断提升。审计、统计、人防、气象等工作进一步加强，广播影视、新闻出版、史志档案、民族宗教、外事侨务、慈善事业等都取得新成效，妇女、未成年人、老年人和残疾人合法权益得到保障。大力开展国防教育，扎实做好双拥工作。驻漯解放军、武警消防官兵和民兵预备役在支持地方经济建设、维护社会稳定等方面作出了重要贡献。

各位代表!2010年各项目标的顺利实现，标志着"十一五"规划预期目标的胜利完成。回首"十一五"，国际金融危机冲击，多种自然灾害频发，资源环境约束趋紧，发展形势复杂多变。面对压力和挑战，我们在市委的坚强领导下，积极应对，科学决策，团结拼搏，攻坚克难，经济社会发展取得了显著成就。

过去的五年，是发展速度明显加快、综合实力显著增强的五年。全市生产总值是"十五"末的2.1倍，人均生产总值由1.3万元增加到2.7万元，全部工业增加值由188亿元增加到452.7亿元，社会消费品零售总额由95.4亿元增加到216亿元，全社会固定资产投资累计突破1300亿元，增长2.4倍。累计财政总收入210.7亿元、地方财政一般预算收入100亿元，均比"十五"时期实现翻番。

过去的五年，是农业基础不断巩固、农村面貌日新月异的五年。粮食产量连创新高，总产累计900万吨。畜牧业产值占农业总产值的比重达到50%，成为全省畜牧强市。蔬菜种植面积发展到85.6万亩，有机蔬菜种植面积全省第一。农业标准化取得新成效，我市成为全国农业综合标准化示范市。全市有效灌溉面积达到225.6万亩，占耕地面积的91%。农业机械化水平大幅提高，农业机械总动力达到250万千瓦。新建改造农村公路2190公里，路网密度居全省第三位。新农村建设试点村达到300个，新解决农村57万人的饮水安全问题，发展户用沼气池20.5万座，开通班车的行政村达98%，"万村千乡市场工程"、"有线电视村村通工程"实现全覆盖。五年发放强农惠农补贴资金29亿元，惠及农户47万户。

过去的五年，是城市建设加快推进、城市形象大幅提升的五年。城市规划面积扩大到100平方公里，市区建成区由48.6平方公里扩大到60平方公里，常住人口由46万人增加到60万人。城市功能不断完善，新建改造一批市区道路、桥梁、供水、供气、供热、公厕等基础设施，污水日处理能力达到20万吨，垃圾无害化处理率85%。城市环境明显改善，城市绿化覆盖率35.2%、绿地率31.3%、人均公共绿地面积12.5平方米，澧河水质一直保持在二类标准。沙澧河开发建设取得阶段性成果，滨河水城的独特魅力已经彰显。我市相继获得中国优秀旅游城市、国家森林城市、省级文明城市等称号，城市环境综合整治定量考核连续五年位居全省前列。漯河城市品位的提升和城市面貌的变化，越来越受到外界的好评，全市人民的满意度越来越高。

过去的五年，是改革开放加快推进、发展活力持续增强的五年。国企改革深入推进，双汇国有产权成功转让，银鸽股权分置改革与重组顺利实施，一批企业通过战略重组焕发生机与活力。政府机构改革取得新成效，高效行政服务机制建设实现新突破。投融资体制和金融体制改革进展顺利，金融生态环境不断优化，我市摘掉了金融高风险区的帽子。创新投融资模式，成功组建城投、发投等投融资平台，在全省第一家发行城投债券，漯河商业银行挂牌运行。农村土地流转、集体林权制度改革走在全省前列，户籍制度改革力度加大，粮食、供销、财政、交通、城建、教育、卫生等方面改革，都有新的进展。对外开放进一步扩大。全市实际利用外资累计达10.8亿美元、是"十五"期间的4.2倍，8家世界500强企业在我市投资发展。对外贸易不断扩大，进出口总额累计达12亿美元。活力漯河更有活力，开放的漯河更加开放。

过去的五年，是社会和谐进步、人民群众得到更多实惠的五年。累计新增城镇就业16万人，安置下岗人员再就业6万人，农村劳动力转移就业50万人，我市荣获首批"中国创业之城"称号。社会事业长足发展，公共服务体系进一步完善，社会保障体系不断健全并向农村延伸。城乡居民收入明显提高，城镇居民人均可支配收入、农民人均纯收入分别比"十五"末增长83.3%和82.9%。社会大局和谐稳定，我市荣获全国社会治安综合治理优秀市、全省平安建设先进市、信访工作先进市称号。

各位代表!过去五年的发展令人鼓舞，漯河的经济实力跨上了一个大台阶，一批拉动经济增长的新板块、新亮点正在形成，广大市民的自豪感和荣誉感不断增强，全市上下干事创业的热情空前高涨。这标志着漯河进入了一个基础更加稳固、环境更加优化、空间更加广阔、动力更加强劲的黄金发展期。这些成绩的取得，是省委、省政府和市委正确领导的结果，是人大、政协监督支持的结果，是全市广大干部群众群策群力、艰苦奋斗的结果。在此，我代表市人民政府，向工作在各条战线的工人、农民、知识分子、干部，向驻漯部队、武警消防官兵、公安民警以及各界人士，致以崇高的敬意!向所有关心支持漯河发展的同志们、朋友们，表示衷心的感谢!

各位代表!五年的奋斗令人难忘，我们在困难中实现了快速发展，在危机中加快了转型步伐，在奋进中坚定了必胜信心，在实干中铸就了新的辉煌。我们深深体会到:只要牢牢把握发展要务，坚持高端定位、坚持工业强市、坚持不懈开放;只要紧紧抓住战略机遇，善于审时度势、善于乘势顺势、善于发挥优势;只要坚持弘扬实干精神，勇于攻坚克难、勇于开拓创新、勇于担当责任;只要时时刻刻牢记宗旨意识，念念不忘体恤民情、尊重民意、改善民生，我们就一定能够推动漯河经济社会跨越发展，就一定能够实现漯河城乡面貌重大变化，就一定能够开创漯河更加美好的未来!

各位代表!在肯定成绩的同时，我们必须清醒地认识到，发展中还有很多限制条件和不足，还会遇到一些深层次矛盾和问题。"十二五"建设成一个什么样的漯河，取决于我们破难攻坚、跨越障碍的胆略和气魄。从这个意义上说，我们还面临着"五大考验"。一是如何加快经济发展方式转变，构建现代产业体系。我市经济总量还不大，综合实力还不强;产业整体层次不够高，第三产业发展滞后，自主创新能力比较弱，资金、土地和环境等要素约束日益加剧。要保持经济又好又快发展，难度增大。二是如何加快拓展城市发展空间，打造更加富有竞争力的时尚之都。我们的城市区域开发起步较晚，产业支撑能力还不够强;城市特色还不够鲜明，城市功能和公共服务相对滞后，城市管理还没有实现精细化，城市文明程度还需要进一步提高。三是如何挖掘优势文化资源，创新漯河文化旅游品牌。许慎文化等历史文化资源挖掘提升不够，食品文化、水韵文化、休闲文化等现代文化资源缺乏有效整合，农业游、工业游和红色游规模较小，没有真正形成文化旅游品牌。四是如何加快推动全民创业，确保城乡居民收入增长位居全省前列。全民创业的氛围还不够浓，指导、培训等服务水平有待提高;农民增收渠道还不够宽，城镇居民收入低于全省平均水平，要大幅度提高城乡居民收入，压力巨大。五是如何强化公共安全和民生改善，建设更加平安向上的和谐漯河。社会事业欠账较多，城乡发展不平衡，群众上学难、看病难等民生问题依然突出;社会安全稳定的基础仍较薄弱，安全稳定隐患在一些领域依然存在。面对考验，我们必须勇于迎难而上、敢于排难奋进、善于克难制胜，努力在发展中破解难题、在发展中寻求突破、在发展中实现跨越。

折叠编辑本段二、今后五年的发展目标和主要任务

今后五年，政府工作的总体要求是:按照市委关于"十二五"的战略部署，以科学发展为主题，以加快转变经济发展方式为主线，以建设中原经济区为重大机遇，以"三化"协调发展为基本途径，以富民强市为中心任务，围绕"一区两城一中心"发展定位，着力调整优化经济结构，着力统筹城乡协调发展，着力深化改革开放，着力保障和改善民生，努力保持经济又好又快发展和社会大局和谐稳定。

"十二五"时期我市经济社会发展的主要目标是:到2015年，力争实现"五个翻番、一个高于、一个同步":经济总量突破1300亿元，地方财政一般预算收入突破50亿元，规模以上工业增加值突破800亿元，服务业增加值突破240亿元，全社会固定资产投资突破800亿元，均比2010年翻一番;主要经济指标增幅高于全省平均水平;城乡居民收入与经济发展同步增长，各项社会事业更加繁荣。

实现上述目标，重点抓好四大任务:

(一)加快提升产业竞争力，致力打造"实力漯河"。按照"做优一产、做强二产、做大三产"的发展思路，全面推动产业优化升级，进一步壮大经济实力，不断增强我市的综合竞争力。继续坚持工业强市战略不动摇，壮大特色主导产业，提升优势传统产业，培育战略新兴产业，推动产业集聚发展，努力打造全省工业强市。到"十二五"末，重点工业企业累计完成投入1000亿元以上，工业企业销售收入突破3000亿元，产业集聚区入驻企业1000家以上、实现主营业务收入2500亿元;全市销售收入超亿元企业发展到200家以上、超10亿元企业20家以上，努力形成"千亿双汇、百亿银鸽"等骨干企业强劲引领、中小企业竞相发展的格局。大力发展现代农业，不断加大农业投入，突出建设现代农业示范基地、优质粮食生产基地、标准化规模养殖基地、无公害蔬菜种植基地，形成装备现代化、生产集约化、管理科学化、服务社会化的现代农业产业体系，努力把我市打造成全省最有影响的现代农业示范市。加快建设区域性综合交通枢纽和现代商贸物流中心，突出发展现代物流、商贸流通、餐饮服务、休闲娱乐、商务会展等产业，逐步形成大商贸、大物流格局。

(二)加快推进改革创新，致力打造"活力漯河"。发扬敢为人先、敢趟深水、敢闯敢试的优良传统，让解放的思想更解放，再掀开放新热潮。深化企业改革，积极推进重点企业战略重组，创造条件支持企业上市。继续推进行政管理体制改革，不断深化财税、金融等重点领域和关键环节的改革，着力构建充满活力、富有效率的科学发展体制机制。加快服务型政府建设，使漯河成为中原地区审批事项少、办事效率高、发展环境好的城市。高起点承接产业转移，着力构建全方位、多层次、宽领域的开放新格局。

(三)加快建设"双百"城市，致力打造"魅力漯河"。坚持高端规划，打造精品建筑，实施精细管理，培育城市精神，在更大格局、更高层次上，奋力拓展城镇发展新空间，坚定不移把漯河建设成为功能完善、绿色宽畅、特色鲜明、宜居宜业、品位时尚的城市。加快推进沙澧河开发建设，加快推进中心城区、县城、中心镇和农村新型社区建设，加快推进经济技术开发区、漯河新区、西城区建设，加快推进"数字漯河"建设。支持发展物联网。到"十二五"末，中心城区基本形成100平方公里、100万人口的城市框架，辐射力和吸引力显著增强，真正把漯河建成中原地区最具魅力的城市。

(四)加快构建和谐社会，致力打造"幸福漯河"。强力推进基本公共服务均等化，逐步形成较为完善的社会服务体系。建设经济适用房100万平方米、廉租房和公租房100万平方米，解决市民住房难问题;改造提升县区和乡镇卫生院，解决群众就医难问题;强力推进养老保险，解决城乡居民养老问题。更加注重优化教育资源布局，投资10亿元在市区新建中小学20所、改扩建80所，全市新增各类幼儿园300所，基本解决义务教育、学前教育发展不均衡问题。推进民主法制，维护公平正义。探索创建"幸福漯河"模式，让全市人民充分享受居住环境更舒适、就学就医更方便、城乡养老全覆盖的幸福生活。

各位代表!《漯河市国民经济和社会发展"十二五"规划纲要(草案)》及说明已印发，请予审议。完成"十二五"规划的目标任务，是政府义不容辞的责任，也是全市人民的共同期盼。只要我们万众一心、众志成城，就一定能如期实现既定目标，就一定能把漯河建设成为一个有实力、有活力、有魅力、人民幸福安康的城市!

折叠编辑本段三、2011年发展目标和重点工作

2011年是我市"十二五"发展的开局之年，我们必须以更加有效的工作，确保开好局、起好步。今年，经济社会发展的主要预期目标是:生产总值增长12%;规模以上工业增加值增长16%;地方财政一般预算收入增长12%;全社会固定资产投资增长20%;社会消费品零售总额增长16%;实际利用外资增长18%，出口总额增长13%;城镇居民人均可支配收入增长10%,农民人均纯收入增长10%;城镇化率提高2个百分点以上;单位生产总值能耗、人口自然增长率、城镇登记失业率均控制在省定目标以内。

实现上述目标，必须着重做好以下六个方面的工作:

(一)围绕招商引资第一任务，倾力打造开放高地。注重用战略眼光谋划开放，用开放寻求新的动力。以更宽的视野拓展开放领域。坚定不移地扩大金融保险、商贸物流、文化旅游等服务业对外开放，加快城乡建设、现代农业、社会事业等领域开放步伐，鼓励民间资本参与投资。积极促进区域合作，全方位、多渠道引进人才、资金、技术和先进的管理经验，鼓励外来资金投向高新技术产业和循环经济产业。扩大出口产品规模，在继续抓好肉类、蔬菜、纸制品等传统产品出口的同时，积极鼓励企业走出去，带动设备和原材料出口，力争外贸出口达到1.9亿美元。以拼抢的劲头抓好招商引资。围绕国家和省"十二五"重点项目专项规划，加强与上级有关部门对接，全年争取上级资金7亿元以上。高水平举办第九届中国(漯河)食品博览会，持续开展一系列招商引资推介活动，力争新签约项目投资总额500亿元以上，其中超亿元项目60个以上、超5亿元项目6个以上，完成30家重点企业的战略重组，引进战略投资30亿元以上。大力开展产业集聚区招商，每个产业集聚区引进投资超亿元以上的工业项目10个以上，引进资金50亿元以上;注重与有影响力的招商代理机构、商会合作，推行委托招商;积极探索"招商大使"专业招商，深化以商招商、以企招商，推进园区共建。以一流的服务优化开放环境。把产业集聚区作为开放主平台，加快完善路网、电网、信息网等基础设施，增强承载配套能力。支持经济开发区申报出口加工区，努力建成功能现代、产业高端、总部集聚的开放先导区和示范区。改善安商环境，严厉打击侵害客商利益和影响企业生产经营的行为。制定相关政策，支持发展总部经济，吸引境内外研发中心入驻。强化公共服务，提高城市综合服务能力，营造良好的投资环境。

(二)围绕做大做强工业，强力推进产业集聚集约发展。突出工业主导地位，致力推动主导产业高端化、传统产业品牌化、新兴产业规模化。全力以赴加快产业集聚区建设。大力实施产业集聚区投资倍增计划，积极开展"培育百亿元产业集聚区、提升产业集聚区基础设施、争创新型产业集聚区"三项活动，力争全市产业集聚区工业项目完成投入200亿元以上，3个产业集聚区主营业务收入均超100亿元、综合排序进入全省前50名。完善管理机制，强化配套服务，统筹解决好产业集聚区内职工食宿、购物、休闲、公共交通等问题。集中精力开展项目建设年活动。全年安排重点项目120个，完成投资150亿元以上。重点抓好双汇5000万只肉鸡、银鸽生活纸和特种纸、恒安闽商产业园、永银盐化工、香港协鑫单晶硅等工业项目，加快推进华电二期、可口可乐二期、金大地三期等重大项目的前期工作。深入开展项目建设观摩点评活动，完善联审联批联办制度，大力开展项目建设环境集中整治，抓好项目跟踪问效。持之以恒壮大特色优势产业。实施大产业、大集团发展战略，培育壮大双汇、乐天澳的利等现有企业，引进国内外食品行业龙头企业。加快建设中部地区重要的造纸基地和全省盐化工基地。振兴纺织、冶金、制革制鞋、机械制造等传统产业。积极培育新兴产业，规划建设电子工业园。着力培育规模企业群体。实施"亿元企业群体培育工程"，新增一批亿元企业。持续开展服务企业活动，做好中小企业全程公共服务平台的应用推广，完善担保体系和信用体系，积极开展银企对接，重点破解融资和用地等难题，最大限度地支持中小企业发展。积极开展上市后备企业培育，新纳入上市后备企业20家以上。加快工业化、信息化融合发展，不断提高企业自主创新能力。

(三)围绕农业增效农民增收，全力夯实"三农"基础。全面落实强农惠农政策，坚持财政支出重点向农村倾斜，确保用于农业农村的总量、增量均有提高。大力发展现代农业。加快粮食生产核心区建设，粮食总产稳定在160万吨以上。以城市近郊和基础较好的乡镇为重点，建成10个市级现代农业示范园区，蔬菜种植面积达到88万亩，完成林业生态工程建设5.3万亩。深入推进"双汇产业化工程"，新发展标准化规模养殖场(小区)20个，着力培育农业产业化龙头企业，积极发展农民专业合作社和行业协会等专业合作组织，大力推行农业标准化，切实加强农产品质量安全监管。不断改善农村生产生活条件。狠抓农田水利基本建设，加快中低产田改造和高产田巩固提升，提高农业防灾减灾能力。新建一批综合水平较高的示范村。新建沼气服务网点20个、户用沼气池1万座，加快推进"农村安全饮水工程"，新解决7万人安全饮水问题。加大对农民工的培训力度，新增培训转移农村劳动力2万人。继续实施"家电下乡"、"摩托车下乡"。加快创建"玉米收获机械化示范市"，机收率达到75%以上。深化农村各项改革。创新农村投融资机制，积极发展村镇银行、小额贷款公司，解决农民贷款抵押难、担保难问题。深入推进集体林权制度改革，抓好全国平原林改示范区试点工作。大力推进农村土地流转。加快推进户籍制度改革，切实解决进城人员就业、就学、住房、养老等实际问题。大力发展县区域经济。完善落实促进县区域经济和乡镇经济发展的政策措施，深入开展争创强县区、强乡镇活动，促进各县区、各乡镇竞相发展。

(四)围绕城乡协调发展，致力加快城镇化进程。实施"城乡建设三年大提升行动"，协调推进市区、县城和小城镇建设。努力构建大城区、大交通格局。按照"科学布局、合理分区、功能完备"的原则，完成漯河新区总体规划编制工作，实施一批重点基础设施建设工程。加快西城区汽车站、高铁客运站、环城水系及相关道路建设，抓好107国道漯河段升级改造工程，改造县乡公路和通村公路100公里，加强交通场站建设，改造漯河汽车中心站，新建一批停车场点。抓好许漯城际轨道交通项目前期工作，石武客专、漯阜铁路建成通车，孟平复线电气化改造工程开工建设。着力提升城市品位。高标准完成沙河二期工程，开工建设沙河三期、澧河二期工程，启动双汇生态水城、沙澧河交汇处等重点区域开发建设。加快建设"梦幻沙澧"河流剧场、香陈湾田园度假社区和凤凰岛生态休闲旅游度假区项目，逐步形成"三点一线"的水上旅游线路，努力把沙澧河风景区建成国家4A级旅游景区。持续强化城市管理，严厉打击违法建设，抓好国家环保模范城市创建工作，实施城市精细化管理，开展市容市貌整治行动。
